# Papanicolaou test in Brazil: analysis of the National Health Survey of 2013 and 2019

**DOI:** 10.11606/s1518-8787.2023057004798

**Published:** 2023-09-14

**Authors:** Gulnar Azevedo e Silva, Giseli Nogueira Damacena, Caroline Madalena Ribeiro, Luciana Leite de Mattos Alcantara, Paulo Roberto Borges de Souza, Célia Landmann Szwarcwald

**Affiliations:** I Universidade do Estado do Rio de Janeiro Instituto de Medicina Social Hesio Cordeiro Rio de Janeiro RJ Brazil Universidade do Estado do Rio de Janeiro. Instituto de Medicina Social Hesio Cordeiro. Rio de Janeiro, RJ, Brazil; II Fundação Oswaldo Cruz Instituto de Comunicação e Informação Científica e Tecnológica em Saúde Rio de Janeiro RJ Brazil Fundação Oswaldo Cruz. Instituto de Comunicação e Informação Científica e Tecnológica em Saúde. Rio de Janeiro, RJ, Brazil; III Ministério da Saúde Programas de Rastreamento Rio de Janeiro RJ Brazil Ministério da Saúde. Programas de Rastreamento. Rio de Janeiro, RJ, Brazil; IV Universidade Federal do Rio de Janeiro Programa de Engenharia Biomédica Rio de Janeiro RJ Brazil Universidade Federal do Rio de Janeiro. Programa de Engenharia Biomédica. Rio de Janeiro, RJ, Brazil

**Keywords:** Cervix Uteri, Neoplasms, Health Services Accessibility, Mass Screening

## Abstract

**OBJECTIVES:**

To compare the coverage of cervical cancer screening in Brazil in 2013 and 2019, investigating the factors associated with having the test performed and the reasons given for not doing it. Additionally, a comparison is made concerning the time taken to receive the test result in SUS (Sistema Único de Saúde) and in the private health services.

**METHODS:**

Using data from the National Health Survey (*Pesquisa Nacional de Saúde* - PNS), prevalence rates and corresponding confidence intervals were calculated to determine the frequency of recent cervical cancer screenings among women aged between 25 and 64 years old in Brazil, for both 2013 and 2019. Poisson regression models were employed to compare the prevalence of the outcome according to sociodemographic characteristics. The reasons for not having the test and the time between performing and receiving the result were also analyzed.

**RESULTS:**

The findings revealed an increase in the coverage of preventive cervical cancer exams in Brazil from 78.7% in 2013 to 81.3% in 2019. Additionally, there was a decline in the proportion of women who had never undergone the exam, from 9.7% to 6.1%. Prevalence of test uptake was higher among white women, those with higher levels of education and income, and those residing in the South and Southeast regions of the country. The most commonly cited reasons for not taking the test were the impression it was unnecessary (45% in both 2013 and 2019) and never having been asked to undergo the test (20.6% in 2013 and 14.8% in 2019).

**CONCLUSIONS:**

Despite the high coverage of screening achieved in the country, there is great inequality in access to the test, and a non-negligible number of women are at greater risk of dying from a preventable disease. Efforts must be made to structure an organized screening program that identifies and captures the most vulnerable women.

## INTRODUCTION

Cervical cancer is a potentially preventable disease primarily caused by persistent infection with the human papillomavirus (HPV)^1^. Employing screening, it is possible to detect precursor lesions that, if treated promptly, prevent the progression to malignant neoplasms.

The most widely utilized screening method worldwide is the Papanicolaou test, which involves the microscopic examination of collected material from the ectocervix and endocervix to identify cellular abnormalities indicative of precursor lesions or cancer. Women with abnormal test results should be referred for further diagnostic investigation, and if the presence of a lesion is confirmed, receive timely treatment^2^.

Countries that have implemented screening programs have witnessed significant reductions in both cervical cancer mortality and incidence rates^3^. The effectiveness of screening relies on achieving widespread coverage within the target population and ensuring appropriate follow-up and treatment for all women whose test results are abnormal^2^.

In Brazil, cervical cancer screening was initiated in the late 1980s and has since followed an opportunistic model. According to the National Guidelines for screening, women aged 25 to 64 are advised to undergo screening every three years^4^.

As part of a strategy to eliminate cervical cancer, the World Health Organization (WHO) recommends that 70% of women over 35 years old be screened^2^. Despite the high estimated coverage reported in national surveys, at 78.8% nationwide^5^ and 80% in major cities^6^, Brazil continues to experience high incidence and mortality rates for this cancer type when compared to other countries^7^.

Conversely, countries like Norway, Finland, Denmark, and Sweden, which initiated screening programs in the 1960s, have witnessed a significant decline in the incidence of cervical cancer, leading to its classification as a rare disease^8^. The effectiveness of screening programs can be enhanced through organized approaches where the target population is identified and regularly invited for testing, as observed in European countries and in contrast to what currently takes place in Brazil and other Latin American nations^3^.

The poorest regions of Brazil, particularly the North and Northeast, exhibit the highest mortality rates attributed to cervical cancer^9,10^. Over the course of four decades, a downward trend has been observed nationwide, except for the rural areas in the North region^11^. While the decline in mortality rates can be largely attributed to screening efforts^12^, the decline rate in Brazil still lingers behind when compared to other countries, such as Chile^13^.

Existing literature has highlighted the disparities in access to cervical cancer screening within the country, with associated factors including individual characteristics like values, beliefs, fear, and limited knowledge about the disease, as well as socioeconomic factors such as income and education, and healthcare-related aspects like ease of scheduling, distance, and the embracement by healthcare providers^14^.

In Brazil, the results of the 2013 National Health Survey (PNS) revealed that the highest proportion of respondents who had undergone cervical cancer screening in the past three years resided in the Southeast and South regions, were of white ethnicity, and had a higher level of education^5^. Conversely, in addition to having the highest mortality rates, the North region also exhibited the lowest estimated screening coverage^17^ and the highest proportion of women who had never undergone the preventive test^18^. Consequently, there is significant inequality in the risk of cervical cancer-related mortality among economically disadvantaged women in the country. It is crucial to understand the screening coverage, factors associated with not undergoing the test, and the profile of the women who remain excluded from screening and tracking initiatives.

This study aims to analyze the coverage and characteristics of cervical cancer screening among women with and without access to private health plans in Brazil in 2013 and 2019.

## METHODS

### Study design

This is a panel study based on data from two editions of the PNS carried out in 2013 and 2019. PNS is a nationwide household-based survey conducted by the Ministry of Health in collaboration with the Brazilian Institute of Geography and Statistics (Instituto Brasileiro de Geografia e Estatística - IBGE) during those respective years. Ethical approval for the PNS was obtained from the National Research Ethics Committee (Comissão Nacional de Ética em Pesquisa, Conep) in July 2013 (approval number: 328,159) for the 2013 edition and in August 2019 (approval number: 3,529,376) for the 2019 edition.

### Sampling

The PNS is part of the IBGE’s Integrated Household Survey System and uses a subsample of the Institute’s Master Sample. The surveyed population consisted of residents of permanent private households in Brazil, excluding those located in special census tracts^19^. The primary units of the Master Sample were stratified based on four criteria: administrative, geographic, location (urban and rural), and statistical, the latter of which further subdivided the previous criteria into homogeneous strata, considering information on total household income and the number of private households.

The sampling plan for both PNS editions utilized a three-stage conglomerate sampling design (census sectors or composition of sectors, households, individuals) with stratification of the primary sampling units according to the Master Sample. Sampling units were selected through simple random sampling in all three stages. In the 2013 edition, a total of 60,202 individuals aged 18 or above were selected for individual interviews, while 85,854 were selected to participate in the 2019 edition.

Expansion factors were calibrated, taking into account population projections for Brazil and its Federation Units. To enable comparisons between the 2013 and 2019 editions of the PNS, the expansion factors of the PNS-2013 were recalibrated, considering the revision of the Population Projection of the Federation Units by Sex and Age from 2010 through 2060. The same population projection was used to calibrate the weights for PNS-2019. For this study, however, only data from individuals aged 18 or above were utilized, totaling 88,943 respondents.

Detailed information about the PNS sampling plan and the calculation of expansion factors can be found in previous publications^20,21^.

### Study variables and data analysis

This study used information from women who responded to the individual questionnaire, answered by a resident selected with equiprobability among all adult residents of the household. In 2013, 31,845 women participated in the survey, and in 2019, 48,102 women were included.

The outcome considered in this study was the performance of a screening test for cervical cancer by women aged 25 to 64 within the past three years. The prevalence of the outcome and the respective 95% confidence intervals were estimated based on the women’s age ranges (< 25 years, 25 to 64, and ≥ 65 years old). The following question from the questionnaire was utilized: “When was the last time you had a reventive exam for cervical cancer?” 1. Less than 1 year ago; 2. From 1 year to less than 2 years ago; 3. From 2 years to less than 3 years ago; 4. 3 years ago or more; 5. Never undergone screening.

Furthermore, the outcome was analyzed based on the method of payment among women who either had or did not have access to a private plan, including whether they paid directly for the test, it was performed through a health plan or conducted via the National Health Service (Sistema Único de Saúde - SUS).

The prevalence of the outcome and the respective 95% confidence intervals were estimated according to sociodemographic variables, including race/skin color (white, black, brown), level of education (incomplete elementary, complete elementary, complete high school, complete higher education), per capita income in minimum wages (MW; up to 1/2 MW, > 1/2 and ≤ 1 MW, > 1 and ≤ 2 MW, > 2 and ≤ 3 MW, > 3 MW), region (North, Northeast, Southeast, South, and Midwest), home situation (urban, rural), municipality of residence (capital, rest of the state), access to a health plan (yes, no), sexual intercourse in the last 12 months (yes, no), and whether there is a history of pregnancy, even if it did not come to term (yes, no). Poisson regression models were used to compare the prevalence of the outcome according to each of the variables mentioned. Crude prevalence ratios (PR) and their respective 95% confidence intervals were estimated.

The analysis of the time until the availability of the result was performed based on whether the test was conducted within the SUS or outside the SUS network. The proportions of women aged 25 to 64 who underwent the screening test and their respective 95% confidence intervals were estimated according to the time the availability of the result (less than 1 month later, between 1 month and less than 3 months later, 3 months or more, pending, never received, failed to pick it up) for tests conducted within the SUS and outside the SUS network.

To compare prevalences, Pearson’s chi-square test was used, adjusted by the Rao-Scott correction (which accounts for the effect of the sampling plan), and converted into an F statistic tested at a significance level of 5%.

Regarding women aged 25 to 64 who reported never having taken a preventive test for cervical cancer, the percentage distribution and their respective 95% confidence intervals were examined based on the main reason reported for never having taken the test.

In the statistical analysis, the PNS sampling design was taken into account while considering the sample weights and the clustering effect. The Software for Statistics and Data Science22, version 14.0, “survey” module was used.

## RESULTS

The results presented in Table 1 demonstrate the coverage of women in the target population (25 to 64 years old) who underwent the Papanicolaou test within the last three years. In 2013, the coverage was 78.7%, while in 2019, it increased to 81.3%. This increase was statistically significant (p < 0.01). Between the two surveys, there was a decrease in the proportion of women aged 25 to 64 who had never taken the test, from 9.7% in 2013 to 6.1% in 2019. Additionally, there was an increase in the proportion of women in all age groups who underwent the test. In both surveys, it was observed that greater coverage was found in the target population (25 to 64 years) compared to other age groups, as shown in Table 1. It is noteworthy, however, that about 50% of women under 25 or over 64 reported undergoing the Papanicolaou test in the two editions of the PNS.

**Table 1:** Percentage distribution (and respective 95% confidence intervals) of women according to time since they had undergone the cervical cancer screening test, by age group. National Health Survey, 2013 and 2019.

Age range (years)	2013						2019						p-value^a^
	
	In the last three years		Over three years ago		Never undergone screening		In the last three years		Over three years ago		Never undergone screening		
					
	%	95%CI	%	95%CI	%	95%CI	%	95%CI	%	95%CI	%	95%CI	
< 25	51.1	48.4–53.7	2.5	1.9–3.4	46.4	43.7–49.0	53.8	51.1–56.4	4.1	3.1–5.4	42.1	39.5–44.9	0.0127
25–64	78.7	77.8–79.7	11.6	10.9–12.3	9.7	9.0–10.4	81.3	80.6–82.0	12.6	12.0–13.2	6.1	5.7–6.5	< 0.001
≥ 65	48.1	45.5–50.7	29.8	27.6–32.1	22.1	20.0–24.4	49.9	48.1–51.6	37.4	35.7–39.2	12.7	11.6–13.9	< 0.001
Total	70.5	69.5–71.4	12.6	12.0–13.2	16.9	16.1–17.7	72.7	72.0–73.4	15.5	14.9–16.0	11.8	11.3–12.4	

95%CI: 95% confidence interval.

^a^ Pearson’s chi-square test p-value adjusted by Rao-Scott correction comparing the prevalence in the years 2013 and 2019.

The flowchart for carrying out the screening test for cervical cancer among women aged 25 to 64 years old with information from the two editions of the PNS is shown in Figure. There was an increase in the proportion of women who reported having undergone the test in the last three years (78.7% in 2013 and 81.3% in 2019). However, there was a reduction in this proportion among those who had access to a private health plan and underwent the examination through their plan (26.1% in 2013 and 22.7% in 2019). It was also seen that a higher proportion of women reported having paid for the test, both among those who did not have a private health plan (12.8% in 2013 and 18.4% in 2019) and among those who reported having (1.2% in 2013 and 3.4% in 2019).

**Figure:**
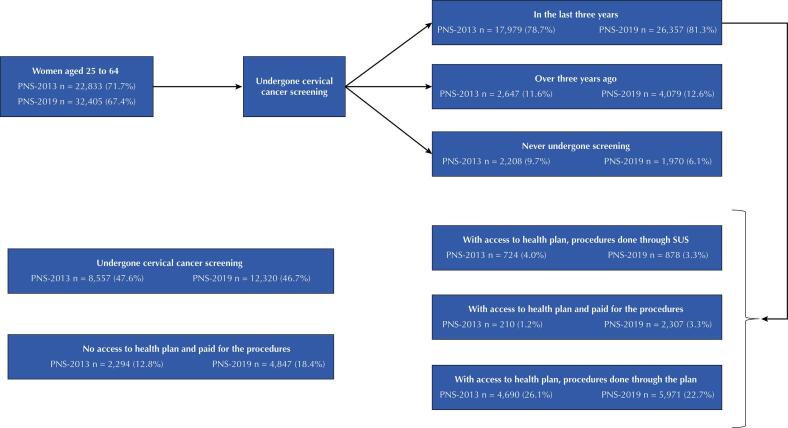
Flowchart of cervical cancer screening in women aged 25 to 64 years. PNS, Brazil, 2013 and 2019.

PNS: *Pesquisa Nacional de Saúde* (National Health Survey).

Note: no information on health plan or how to perform the preventive cervical exam: PNS-2013 n = 1,503 (8.4%) and PNS-2019 n = 34 (0.1%).

The prevalence of women aged 25 to 64 who reported having undergone the test in the last three years in both surveys was higher among white women (82.1% in 2013 and 83.4% in 2019) compared to brown and black women (75.1% and 76.7% in 2013; 79.3% and 81.2% in 2019). It is also observed that the prevalence increases due to better schooling and higher family income. Moreover, women who reported having a private health plan showed higher coverage rates. As for the differences by geographic areas, higher prevalences were observed in the South and Southeast regions, among residents of capitals and urban areas. Conversely, women who had not engaged in sexual intercourse in the last 12 months and those who had never been pregnant exhibited the lowest coverage rates (Table 2).

**Table 2:** Prevalence (and respective 95% confidence intervals) of cervical cancer screening in the last three years prior to the survey among women aged 25 to 64 by socioeconomic variables, geographic location, and access to a health plan. National Health Survey, 2013 and 2019.

Variables	2013				2019			
	
	%	95%CI	PR	95%CI	%	95%CI	PR	95%CI
Ethnicity/skin color								
White	82.1	80.8–83.4	1	–	83.4	82.2–84.5	1	–
Black	76.7^a^	73.2–79.9	0.93	0.89–0.98	81.2^a^	79.2–83.1	0.97	0.95–1.00
Brown	75.1^a^	73.6–76.6	0.91	0.89–0.94	79.3^a^	78.3–80.3	0.95	0.93–0.97
Level of education								
Incomplete elementary	71.4	69.7–73.0	1	–	72.5	71.1–73.9	1	–
Complete elementary	77.1	74.5–79.5	1.08	1.04–1.12	79.6	77.6–81.5	1.1	1.06–1.13
Complete high school	82.7	81.2–84.2	1.16	1.13–1.19	84.2	83.1–85.4	1.16	1.14–1.19
Complete higher education	88.4	81.2–84.2	1.24	1.20–1.28	90.4	89.2–91.5	1.25	1.22–1.28
*Per capita* income								
Up to ½ MW	69.6^a^	67.4–71.7	1	–	73.5^a^	72.0–74.9	1	–
> ½ and ≤ 1 MW	75.9^a^	74.1–77.7	1.09	1.05–1.13	79.9^a^	78.5–81.2	1.09	1.06–1.11
> 1 and ≤ 2 MW	81.4^a^	79.7–82.9	1.17	1.13–1.21	83.7^a^	82.2–85.0	1.14	1.11–1.17
> 2 and ≤ 3 MW	87.7	85.3–89.8	1.26	1.21–1.31	88.2	86.2–90.0	1.2	1.17–1.24
> 3 MW	90.0	88.0–91.7	1.29	1.25–1.34	91.6	89.9–93.0	1.25	1.21–1.28
Region								
North	75.0^a^	72.3–77.6	1	–	79.0^a^	77.2–80.7	1	–
Northeast	74.2^a^	72.5–75.9	0.99	0.95–1.03	76.4^a^	75.2–77.5	0.97	0.94–0.99
Southeast	80.4^a^	78.7–82.0	1.07	1.03–1.12	84.1^a^	82.7–85.3	1.06	1.04–1.09
South	82.6	80.2–84.7	1.1	1.05–1.15	84.8	83.3–86.2	1.07	1.04–1.10
Midwest	80.7	78.8–82.4	1.07	1.03–1.12	78.8	76.6–80.9	1	0.96–1.03
Geographical area								
Urban	79.6^a^	78.5–80.6	1.09	1.05–1.13	82.2^a^	81.4–83.0	1.1	1.07–1.13
Rural	73.0	70.5–75.4	1	–	74.8	73.0–76.4	1	–
Location								
Capital	83.1^a^	81.9–84.2	1.08	1.05–1.10	85.0^a^	84.0–86.0	1.06	1.04–1.08
Rest of the state	77.2^a^	76.0–78.4	1	–	80.1^a^	79.2–81.0	1	–
Health plan								
Yes	89.6	88.1–90.9	1.2	1.18–1.23	91.2	90.1–92.2	1.18	1.16–1.20
No	74.5^a^	73.3–75.7	1	–	77.5^a^	76.6–78.3	1	–
Sexual intercourse in the last 12 months								
Yes	83.6^a^	82.6–84.6	1.35	1.27–1.43	86.0^a^	85.1–86.8	1.31	1.24–1.38
No	62.1	58.3–65.7	1	–	65.9	62.5–69.1	1	–
History of at least one pregnancy								
Yes	82.4	81.2–83.5	1.11	1.07–1.16	82.2	81.4–82.9	1.06	1.04–1.09
No	73.9^a^	71.0–76.6	1	–	77.5^a^	75.7–79.1	1	–

95%CI: 95% confidence interval; MW: minimum wage; PR: prevalence ratio estimated by bivariate Poisson regression.

^a^ p < 0.05 value of Pearson’s chi-square test adjusted by Rao-Scott correction comparing the prevalences in 2013 and 2019.

Furthermore, when comparing prevalences based on sociodemographic and geographic factors between 2013 and 2019, an overall increase in screening test coverage was observed across all regions, except for the Midwest region and among residents in rural areas. However, there was no increase during this period for women with private health plans, those who had not engaged in sexual intercourse in the last 12 months, and those who had already been pregnant.

In 2013, 40.9% of the women who underwent the Papanicolaou test at SUS received their results in less than a month. At this same year, 87.2% of women who had their Papanicolaou test done at private health services reported receiving their results within the specified time frame, with a statistically significant difference compared to those who had the test done at SUS. The same pattern was observed in 2019 (with 39.7% and 91.4%, respectively) (Table 3).

**Table 3:** Percentage distribution (and respective 95% confidence intervals) of women aged 25 to 64 years who underwent the screening test for cervical cancer in the last three years before the survey by time of receipt of the test result according to the test carried out by SUS or non-SUS services, National Health Survey, 2013 and 2019.

How long until the test results were made available?	2013				2019			
	
	SUS^a^		Non-SUS^a^		SUS^a^		Non-SUS^a^	
			
	%	95%CI	%	95%CI	%	95%CI	%	95%CI
Under 1 month later	40.9	39–2–42.7	87.2	86.0–88.4	39.7	38.0–41.3	91.4	90.5–92.3
Between 1 and less than 3 months later	42.0	40.2–43.8	7.8	6.9–8.8	41.4	39.6–43.2	6.1	5.2–7.0
3 months or more	8.7	7.6–10.0	2.3	1.8–3.0	9.8	9.0–10.8	0.4	0.2–0.7
Pending	6.3	5.5–7.3	2.1	1.6–2.7	6.4	5.7–7.1	1.5	1.2–1.8
Never received	1.4	1.1–1.8	0.2	0.1–0.3	1.9	1.6–2.3	0.3	0.1–0.5
Failed to pick up	0.6	0.4–0.8	0.4	0.2–0.6	0.8	0.6–1.2	0.4	0.2–0.5

95%CI: 95% confidence interval.

^a^ The prevalence of SUS and non-SUS rates in each year was compared using Pearson’s chi-square test, adjusted by Rao-Scott correction, resulting in a 95% confidence interval and a significant p-value of < 0.001.

In 2013, only 1.4% of women who had the Papanicolaou test at SUS reported never receiving their results, which was even lower (0.2%) for those who had the test done in the private network, again indicating a statistically significant difference between SUS and non-SUS services (p < 0.001). This significant difference between SUS and non-SUS services was maintained. In 2019, this difference between having taken the exam and not having received the result in the SUS and outside the SUS remained the same (p < 0.001) (Table 3).

The primary reasons cited by participants for not undergoing the exam in both editions of the PNS were consistent, with 45% of women saying “I don’t think it’s necessary.” Another common reason mentioned by 20.6% of women in 2013 and 14.8% in 2019 was “having never been instructed to do so.” Following these reasons, factors such as feeling ashamed or not having had sexual intercourse were reported. In the 2013 survey, 3.8% of women mentioned difficulty scheduling the exam, whereas this reason was not brought up in 2019 (Table 4).

**Table 4:** Percentage distribution (and respective 95% confidence intervals), of the main reported reasons for never having undergone a cervical cancer screening test in the National Health Survey conducted in 2013 and 2019.

Main reason for never having taken the screening test for cervical cancer	2013		2019	
	
	%	95%CI	%	95%CI
Think it unnecessary	45.5	41.8–49.2	45.1	41.6–48.6
Never instructed to take the exam	20.6	17.5–24.1	14.8	12.8–17.1
Is ashamed	9.8	8.0–12.1	13.1	10.8–15.7
Never had intercourse	7.2	5.5–9.3	8.8	6.7–11.4
Other	6.7	4.7–9.5	2.7	1.8–4.0
Had difficulties making an appointment	3.8	2.8–5.2	–	–
Doesn’t know whom to look for or where to go	0.7	0.4–1.1	2	1.1–3.6
The waiting time at the health service is too long	1.6	1.1–2.5	2.7	2.0–3.6
Health service was too far away or had transport difficulties	1.6	0.9–3.0	1.9	1.0–3.5
Facing financial difficulties	1.2	0.8–2.0	2.1	1.4–3.2
The appointment is set, but not done yet	0.7	0.4–1.2	1.4	0.9–2.1
Service opening hours are incompatible with the patient’s work or home activities	0.4	0.2–0.8	2.7	1.8–3.9
Unable to make an appointment through the health plan	-	–	0.5	0.2–1.5
Undergone uterus removal surgery/hysterectomy	-	–	2.3	1.2–4.5

95%CI : 95% confidence interval.

Among women belonging to the target population who reported never having taken the test (9.7% in 2013 and 6.1% in 2019), the highest prevalence was among brown and black women, with lower education and income, higher parity, with no access to health plans, living in the North and Northeast regions, outside the capital, when compared to those who underwent the test in the last three years (data not shown).

## DISCUSSION

Based on self-reported information from the two editions of the PNS, the coverage of cervical cancer screening (Papanicolaou) in Brazil increased between 2013 and 2019 among women aged 25 to 64 (78.7% and 81.3% respectively, p < 0.01). While coverage was higher among white women, residents of the South and Southeast regions, and residents of urban areas or capitals, the most significant increases were observed among black women with lower income (up to two MW), and those without a private health plan. Lower coverage was also noted among women who had not engaged in sexual intercourse in the last 12 months and those who had never been pregnant. The proportion of women paying for the test was higher in 2019, both among those with private health plans and those without.

These findings reinforce the results of national and international studies that have demonstrated an association between race, income, education, and the taking of screening tests. Studies conducted in Belgium and Switzerland have reported an association between income, education level, and completion of screening tests, with differences between individuals who never underwent the test and those who were delayed. The latter group was associated with older age, while never having taken the exam was associated with foreign nationalities^23^. In the United States, an association was also identified between not undergoing the test and younger ages, non-white ethnicity, lower income, and education level^24^.

SUS remains the main responsible for screening in the country. Notably, among women who did undergo the test, there was an increase in those who paid for it, including both exclusive SUS users and individuals with private health plans. While this finding may indicate challenges in accessing the test at SUS or at the private sector, it also reflects an increased awareness of the need for screening. On the other hand, the rise in test performance among individuals under 25 years old in the last three years emphasizes the need to strengthen clinical guidelines that define the target population as those aged 25 to 64 years^4^.

Given that screening is conducted opportunistically in the country, without actively recruiting women in the target age group, it is expected that seeking prenatal care would increase the number of women receiving the Papanicolaou test, taking advantage of the contact with health services. Indeed, in 2013 and 2019, higher coverage was observed among women who had been pregnant at least once in their lifetime, highlighting that prenatal care provides an opportunity for doing the test. However, the opportunistic model does not facilitate the identification of unscreened women or the follow-up of those with abnormal screening results^25^.

Among the primary reasons for not undergoing the test, as described in the literature, lack of knowledge about the test’s importance, fear, shame, and difficulties in accessing health services arise as prominent factors^14^. In this study, the primary reason for not undergoing the test was not considering it necessary (45.1%), followed by the lack of guidance to do so (14.8%). These findings highlight the need for investments in health education, particularly among women with lower income and education levels, as this group represents the highest proportion of individuals who have never taken the exam. For a successful screening program, effective communication strategies and outreach efforts targeting women in the target age group are crucial to enhance adherence.

Additionally, organizing and ensuring the availability of diagnostic investigations and treatment in the healthcare network are essential steps to ensure the full success of screening programs. Delays in making test results available can lead to disinterest and dissatisfaction, and hinder proper follow-up for women with abnormal findings^25^. Furthermore, the quality of the screening exam plays a vital role in identifying precursor lesions of cervical cancer.

One quality criterion, as observed by the National Quality Program in Cytopathology (Programa Nacional de Qualidade em Citopatologia), is the timely release of reports by the laboratory within 30 days of receiving the samples^26^. Both in 2013 and 2019, the prevalence of preventive test reports received within 30 days was about twice as high among women who underwent the test through the health plan compared to those who took it through the SUS network. This finding exposes the challenges and lack of coordination within the SUS healthcare services, hindering the program’s effectiveness^27^.

The opportunistic screening model and inadequate follow-up prevent achieving the same impact as other Latin American countries, such as Chile^13^. In São Paulo, the implementation of an organized screening program in a municipality significantly increased coverage and the identification of cancer cases in their early stages^28^. International experiences confirm this finding, such as the experience of Slovenia, which has carried out opportunistic screening actions for cervical cancer since the 1960s, and, by investing in the implementation of a national screening program at the end of the 1990s, perceived a reduction of approximately 40% in its incidence from 2003 to 2009^29^.

The organization of screening also implies the reduction of inequalities in access. In European countries, socioeconomic status was associated with participation in opportunistic screening but not observed in organized programs^30^. In the United States, the cancer control program developed by the Center for Disease Control prioritized women who had never taken the exam or were overdue. The initial results of the program showed a nearly twofold higher rate of precursor lesions and cancer identified in this group compared to women who were regularly screened^31^.

Some limitations of the study should be noted. The information used was collected in an interview based a questionnaire structured in two cross-sectional surveys based on self-reports by the interviewees. which may be subject to memory bias. However, these surveys were conducted with scientific rigor to ensure the quality and reliability of the information^32^. The generalization of PNS data is considered safe^33^ and has contributed to the planning and monitoring of health actions in the country.

In Brazil, the implementation of a screening program with an active call to the target population and comprehensive monitoring of actions should be a priority in cancer control policies. It is crucial to include women from the most vulnerable segments of the population, as they are at the highest risk of dying from a preventable disease.

The results of this study show that the coverage of cervical cancer screening in Brazil, despite being relatively high, still expose inequality in access and, mainly, in receiving the result as a function of socioeconomic level, skin color, and access to a private health plan healthcare. Despite increased access among these groups, several obstacles must be overcome to achieve the expected impact on cervical cancer morbidity and mortality.
