# Supplementary material for: Papanicolaou test in Brazil: analysis of the National Health Survey of 2013 and 2019
Source: Rev Saude Publica. 2023 Sep 14;57:55. doi: 10.11606/s1518-8787.2023057004798 (PMC10519687; doi:10.11606/s1518-8787.2023057004798)
Supplement: Translation

# Translation: Exame de Papanicolaou no Brasil: análise da Pesquisa Nacional de Saúde em 2013 e 2019

**Authors:** Gulnar Azevedo e Silva, Giseli Nogueira Damacena, Caroline Madalena Ribeiro, Luciana Leite de Mattos Alcantara, Paulo Roberto Borges de Souza, Célia Landmann Szwarcwald

**Affiliations:** I Brazil Universidade do Estado do Rio de Janeiro. Instituto de Medicina Social Hesio Cordeiro. Rio de Janeiro, RJ, Brazil; II Brazil Fundação Oswaldo Cruz. Instituto de Comunicação e Informação Científica e Tecnológica em Saúde. Rio de Janeiro, RJ, Brazil; III Brazil Ministério da Saúde. Programas de Rastreamento. Rio de Janeiro, RJ, Brazil; IV Brazil Universidade Federal do Rio de Janeiro. Programa de Engenharia Biomédica. Rio de Janeiro, RJ, Brazil

**Keywords:** Colo do Útero, Neoplasias, Acesso aos Serviços de Saúde, Programas de Rastreamento

## Abstract

**OBJETIVOS:**

Comparar a cobertura do rastreamento do câncer de colo do útero no Brasil em 2013 e 2019, investigar os fatores associados à realização do exame e os motivos informados para não ter realizado, além de comparar o tempo do recebimento do resultado do exame no SUS e na rede privada.

**MÉTODOS:**

A partir de dados da Pesquisa Nacional de Saúde (PNS) foram calculadas as prevalências e os respectivos intervalos de confiança de realização do exame preventivo do câncer do colo do útero há menos de três anos, em mulheres de 25 a 64 anos, em 2013 e 2019. Modelos de regressão de Poisson foram utilizados para comparar as prevalências do desfecho segundo características sociodemográficas. Também foram analisados os motivos para não ter feito o exame e o tempo entre a realização e o recebimento do laudo.

**RESULTADOS:**

Houve aumento na cobertura do exame preventivo no Brasil entre 2013 (78,7%) e 2019 (81,3%) e redução na proporção de mulheres que nunca fizeram o exame de 9,7% para 6,1%. A prevalência de realização do exame foi maior em mulheres brancas, melhor escolaridade e renda mais alta, residentes nas regiões Sul e Sudeste. Os motivos mais frequentes para não realizar o exame foram achar desnecessário (45% em 2013 e em 2019) e nunca ter sido orientada a fazê-lo (20,6% em 2013 e 14,8% em 2019).

**CONCLUSÕES:**

Apesar das elevadas coberturas de rastreamento alcançadas pelo país, há grande desigualdade no acesso ao exame, e uma parcela não desprezível de mulheres está sob maior risco de morrer por uma doença que pode ser evitada. Esforços devem ser feitos para a estruturação de um programa de rastreamento organizado que identifique e capte as mulheres mais vulneráveis.

## INTRODUÇÃO

O câncer do colo do útero é potencialmente evitável, pois trata-se de uma doença que tem como causa a infecção persistente pelo papilomavírus humano (HPV)^1^ e, a partir de seu rastreamento, é possível identificar lesões precursoras cujo tratamento impede a evolução para neoplasia maligna.

O método de rastreamento mais utilizado no mundo é o exame de Papanicolaou, que consiste na análise microscópica de material colhido da ectocérvice e endocérvice para identificação de alterações celulares que caracterizam lesões precursoras ou câncer. Mulheres com exames alterados devem ser encaminhadas para a investigação diagnóstica, e, quando confirmada a lesão, para o tratamento oportuno^2^.

Em países onde programas de rastreamento foram implementados, observaram-se reduções expressivas na mortalidade e na incidência desse câncer^3^. A efetividade do rastreamento depende de uma alta cobertura na população-alvo, bem como da garantia de seguimento e tratamento adequado de todas as mulheres com exames alterados^2^.

No Brasil, o rastreamento para o câncer de colo do útero em todo o território nacional foi iniciado no fim da década de 1980 e permanece no modelo oportunístico até os dias atuais. As Diretrizes Nacionais para o rastreamento recomendam a realização do exame por mulheres de 25 a 64 anos de idade a cada três anos^4^.

Como estratégia para a eliminação do câncer do colo do útero, a Organização Mundial da Saúde (OMS) recomenda que 70% das mulheres acima dos 35 anos sejam rastreadas^2^. No Brasil, apesar de elevadas coberturas estimadas nos inquéritos nacionais, 78,8% no país^5^ e 80% nas capitais^6^, as taxas de incidência e mortalidade por esse tipo de câncer permanecem altas se comparadas às de outros países^7^.

Em países como Noruega, Finlândia, Dinamarca e Suécia, que iniciaram o rastreamento na década de 1960, a incidência desse tipo de câncer caiu de forma vertiginosa, sendo considerado hoje uma doença rara^8^. O caráter organizado do rastreamento, em que a população-alvo é conhecida e convidada periodicamente para a realização do exame, também confere maior efetividade aos programas, como ocorre em outros países da Europa, quando comparados ao Brasil e outros países da América Latina^3^.

Nas regiões mais pobres do Brasil, Norte e Nordeste, observam-se as mais altas taxas de mortalidade por câncer de colo do útero^9,10^. Ao longo de quatro décadas houve tendência de redução em todo o país, com exceção do interior da região Norte^11^. Embora as curvas descendentes na mortalidade possam ser atribuídas em grande parte ao rastreamento^12^, a velocidade da queda no Brasil foi inferior à observada em outros países, como o caso do Chile^13^.

As desigualdades no acesso ao exame de rastreamento de câncer do colo do útero no país já foram evidenciadas na literatura, e os fatores associados a não realização do exame englobam características individuais, como valores, crenças, medo e desconhecimento sobre a doença; socioeconômicas, como renda e escolaridade; e dos serviços de saúde, como facilidade de agendamento, distância e acolhimento dos profissionais^14^.

No Brasil, os resultados da Pesquisa Nacional de Saúde (PNS) de 2013 mostraram que a maior proporção de entrevistadas que realizaram o exame nos últimos três anos era de residentes nas regiões Sudeste e Sul, brancas e de maior nível educacional^5^. A região Norte, além das maiores taxas de mortalidade, apresenta também as menores coberturas estimadas de rastreamento^17^e maior proporção de mulheres que nunca realizaram o exame preventivo^18^. Há, portanto, grande desigualdade no risco de óbito por câncer de colo de útero entre as mulheres mais pobres do país, sendo fundamental conhecer a cobertura do rastreamento, os fatores associados a não realização do exame e o perfil das mulheres que ainda são excluídas das ações de rastreamento.

Este estudo tem como objetivo analisar a cobertura e as características do rastreamento do câncer de colo do útero entre mulheres com e sem plano de saúde privado no Brasil em 2013 e 2019.

## MÉTODOS

### Desenho do Estudo

Trata-se de um estudo de painel com base nos dados de duas edições da PNS realizadas em 2013 e 2019. Um inquérito de âmbito nacional e base domiciliar, a PNS foi realizada pelo Ministério da Saúde em parceria com o Instituto Brasileiro de Geografia e Estatística (IBGE), nos anos de 2013 e 2019. A PNS teve aprovação da Comissão Nacional de Ética em Pesquisa (Conep) em julho de 2013 sob o número n. 328.159 para a edição de 2013, e em agosto de 2019 sob o número n. 3.529.376 para a edição de 2019.

### Amostragem

A PNS faz parte do Sistema Integrado de Pesquisas Domiciliares do IBGE e utiliza uma subamostra da Amostra Mestra do Instituto. A população pesquisada corresponde aos moradores de domicílios particulares permanentes do Brasil, exceto os localizados nos setores censitários especiais^19^. As unidades primárias da Amostra Mestra são estratificadas segundo quatro critérios: administrativo, geográfico, situação (urbano e rural) e estatístico, sendo que este último subdivide os critérios anteriores em estratos homogêneos, segundo as informações de rendimento total dos domicílios e número de domicílios particulares.

O plano amostral das duas edições da PNS foi por conglomerado em três estágios de seleção (setores censitários ou composição de setores, domicílios, indivíduos), com estratificação das unidades primárias de amostragem de acordo com a Amostra Mestra. Nos três estágios, a seleção das unidades de amostragem foi realizada por amostragem aleatória simples. Na edição de 2013, foram selecionados 60.202 indivíduos de 18 anos ou mais de idade para a entrevista individual e na edição de 2019 foram selecionados 85.854 indivíduos.

Os fatores de expansão foram calibrados levando em consideração as projeções populacionais para o Brasil e Unidades da Federação. Para permitir comparações entre as edições 2013 e 2019 da PNS, nova calibração dos fatores de expansão da PNS-2013 foi feita considerando a revisão da Projeção da População das Unidades da Federação por Sexo e Idade, para o período de 2010–2060. A mesma projeção populacional foi utilizada na calibração dos pesos da PNS-2019. Para este estudo, porém, foram utilizados apenas os dados de indivíduos de 18 anos ou mais de idade, totalizando 88.943 entrevistados.

Informações detalhadas sobre o plano amostral da PNS e o cálculo dos fatores de expansão podem ser encontradas em publicações anteriores^20,21^.

### Variáveis do Estudo e Análise de Dados

Neste estudo, foram utilizadas as informações de mulheres que responderam ao questionário individual, respondido por um morador selecionado com equiprobabilidade entre todos os moradores adultos do domicílio. Participaram da pesquisa em 2013, 31.845 mulheres e, em 2019, 48.102 mulheres.

O desfecho considerado neste trabalho foi a realização de exame preventivo de câncer de colo de útero há menos de três anos por mulheres de 25 a 64 anos de idade. As prevalências do desfecho e os respectivos intervalos de confiança de 95% foram estimados segundo faixa de idade das mulheres (< 25 anos, 25–64 anos e ≥ 65 anos). Utilizou-se a seguinte pergunta do questionário: “Quando foi a última vez que a Sra. fez um exame preventivo para câncer de colo do útero? 1. Há menos de 1 ano; 2. De 1 ano a menos de 2 anos; 3. De 2 anos a menos de 3 anos; 4. Há 3 anos ou mais; 5. Nunca fez”.

Adicionalmente, o desfecho foi analisado segundo forma de pagamento entre as mulheres que tinham ou não plano de saúde, se pagaram diretamente o exame, se realizaram via plano de saúde ou se realizaram pelo Sistema Único de Saúde (SUS).

A prevalência do desfecho e os respectivos intervalos de confiança de 95% foram estimados segundo variáveis sociodemográficas: raça/cor da pele (branca, preta, parda); grau de escolaridade (fundamental incompleto, fundamental completo, médio completo, superior completo); renda per capita em salários mínimos (SM; até 1/2 SM, > 1/2 e ≤ 1 SM, > 1 e ≤ 2 SM, > 2 e ≤ 3 SM, > 3 SM); região (Norte, Nordeste, Sudeste, Sul e Centro-Oeste); situação domiciliar (urbana, rural); município de residência (capital, restante do estado); posse de plano de saúde (sim, não); relação sexual nos últimos 12 meses (sim, não), ficou grávida alguma vez na vida mesmo que a gestação não tenha chegado até o final (sim, não). Modelos de regressão de Poisson foram utilizados para comparar as prevalências do desfecho segundo cada uma das variáveis citadas. As razões de prevalência (RP) brutas e seus respectivos intervalos de 95% de confiança foram estimados.

Foi realizada análise do tempo de recebimento do resultado de acordo com a realização do exame no SUS/não SUS. As proporções de mulheres de 25–64 anos que realizaram o exame preventivo e respectivos intervalos de confiança de 95% foram estimados segundo o tempo de recebimento do resultado (menos de 1 mês depois, entre 1 mês e menos de 3 meses depois, 3 meses ou mais, ainda não recebeu, nunca recebeu, não foi buscar) segundo realização do exame pelo SUS e fora da rede do SUS.

Para a comparação das prevalências, foi utilizado o teste Qui-Quadrado de Pearson ajustado pela correção de Rao-Scott (que leva em consideração o efeito do plano de amostragem) e convertido em uma estatística F, testada no nível de significância de 5%.

Em relação às mulheres da faixa de idade de 25 a 64 que relataram nunca ter feito um exame preventivo de câncer de colo de útero foi investigada a distribuição percentual e seus respectivos intervalos de confiança de 95% segundo principal motivo relatado de nunca terem feito o exame.

Na análise estatística, considerou-se o desenho de amostragem da PNS levando em conta as ponderações amostrais e o efeito de conglomeração. Utilizou-se o *Software for Statistics and Data Science*^22^, versão 14.0, módulo “*survey*”.

## RESULTADOS

Os resultados apresentados na Tabela 1 mostram que a cobertura de mulheres da população-alvo (25–64 anos) que realizaram o exame de Papanicolaou nos últimos três anos foi de 78,7% em 2013 e de 81,3% em 2019, sendo o aumento estatisticamente significativo (p < 0,01). Entre as duas pesquisas houve queda na proporção de mulheres de 25–64 anos que nunca realizaram o exame, de 9,7% para 6,1% e aumento da proporção das que realizaram o exame em todas as faixas etárias. Em ambas as pesquisas, maiores coberturas foram observadas na população-alvo (25 a 64 anos) se comparadas às demais faixas etárias (Tabela 1). Chama atenção, contudo, que cerca de 50% das mulheres abaixo dos 25 anos ou acima dos 64 anos referiram realizar o exame de Papanicolaou nas duas edições da PNS.

**Tabela 1:** Distribuição percentual (e respectivos intervalos de confiança de 95%) de mulheres segundo tempo de realização do exame preventivo de câncer de colo de útero por faixa de idade. Pesquisa Nacional de Saúde, 2013 e 2019.

Faixa de idade (anos)	2013						2019						Valor de p-values^a^
	
	Nos últimos três anos		Há mais de três anos		Nunca fez		Nos últimos três anos		Há mais de três anos		Nunca fez		
					
	%	IC95%	%	IC95%	%	IC95%	%	IC95%	%	IC95%	%	IC95%	
< 25	51,1	48,4–53,7	2,5	1,9–3,4	46,4	43,7–49,0	53,8	51,1–56,4	4,1	3,1–5,4	42,1	39,5–44,9	0,0127
25–64	78,7	77,8–79,7	11,6	10,9–12,3	9,7	9,0–10,4	81,3	80,6–82,0	12,6	12,0–13,2	6,1	5,7–6,5	< 0,001
≥ 65	48,1	45,5–50,7	29,8	27,6–32,1	22,1	20,0–24,4	49,9	48,1–51,6	37,4	35,7–39,2	12,7	11,6–13,9	< 0,001
Total	70,5	69,5–71,4	12,6	12,0–13,2	16,9	16,1–17,7	72,7	72,0–73,4	15,5	14,9–16,0	11,8	11,3–12,4	

IC95%: intervalo de confiança de 95%.

^a^ Valor de p do teste qui-quadrado de Pearson ajustado pela correção de Rao-Scott em comparação das prevalências dos anos 2013 e 2019.

O fluxograma de realização do exame preventivo de câncer de colo de útero entre mulheres de 25 a 64 anos de idade com as informações das duas edições da PNS é apresentado na Figura. Observou-se aumento da proporção de mulheres que informaram ter realizado o exame nos últimos três anos (78,7% em 2013 e 81,3% em 2019). No entanto, houve uma redução dessa proporção entre as que tinham plano de saúde privado e realizaram o exame pelo plano (26,1% em 2013 e 22,7% em 2019). Foi visto, ainda, que uma maior proporção de mulheres referiu ter pago pelo exame, tanto entre as que não tinham plano de saúde privado (12,8% em 2013 e 18,4% em 2019) quanto entre aquelas que referiram ter (1,2% em 2013 e 3,4% em 2019).

**Figura:** Fluxograma de realização do exame preventivo de câncer de colo de útero em mulheres de 25 a 64 anos de idade. PNS, Brasil, 2013 e 2019.

PNS: Pesquisa Nacional de Saúde.

Nota: Sem informação de plano de saúde ou forma de realização do exame preventivo de colo de útero: PNS-2013 n = 1.503 (8,4%) e PNS-2019 n = 34 (0,1%).

A prevalência de mulheres de 25 a 64 anos que informaram ter realizado o exame nos últimos três anos nas duas pesquisas foi maior entre as de cor branca (82,1% em 2013 e 83,4% em 2019) se comparadas às pardas e pretas (75,1% e 76,7% em 2013; 79,3% e 81,2%, em 2019). Observa-se, igualmente, que as prevalências aumentam em função da melhor escolaridade e maior renda familiar. Adicionalmente, maiores coberturas são encontradas entre as mulheres que referiram ter plano de saúde privado. Quanto às diferenças por áreas geográficas, maiores prevalências foram observadas nas regiões Sul e Sudeste, entre as residentes nas capitais e zonas urbanas. As menores coberturas foram apresentadas por mulheres que não tiveram relações sexuais nos últimos 12 meses e por aquelas que nunca engravidaram (Tabela 2).

**Tabela 2:** Prevalência (e respectivos intervalos de confiança de 95%) de exame preventivo de câncer de colo de útero nos últimos três anos anteriores à pesquisa entre mulheres de 25 a 64 anos por variáveis socioeconômicas, de localização geográfica e posse de plano de saúde. Pesquisa Nacional de Saúde, 2013 e 2019.

Variáveis	2013				2019			
	
	%	IC95%	RP	IC95%	%	IC95%	RP	IC95%
Raça/cor da pele								
Branca	82,1	80,8–83,4	1	–	83,4	82,2–84,5	1	–
Preta	76,7^a^	73,2–79,9	0,93	0,89–0,98	81,2^a^	79,2–83,1	0,97	0,95–1,00
Parda	75,1^a^	73,6–76,6	0,91	0,89–0,94	79,3^a^	78,3–80,3	0,95	0,93–0,97
Grau de escolaridade								
Fundamental incompleto	71,4	69,7–73,0	1	–	72,5	71,1–73,9	1	–
Fundamental completo	77,1	74,5–79,5	1,08	1,04–1,12	79,6	77,6–81,5	1,1	1,06–1,13
Médio completo	82,7	81,2–84,2	1,16	1,13–1,19	84,2	83,1–85,4	1,16	1,14–1,19
Superior completo	88,4	81,2–84,2	1,24	1,20–1,28	90,4	89,2–91,5	1,25	1,22–1,28
Renda *per capita*								
Até ½ SM	69,6^a^	67,4–71,7	1	–	73,5^a^	72,0–74,9	1	–
> ½ e ≤ 1 SM	75,9^a^	74,1–77,7	1,09	1,05–1,13	79,9^a^	78,5–81,2	1,09	1,06–1,11
>1 e ≤ 2 SM	81,4^a^	79,7–82,9	1,17	1,13–1,21	83,7^a^	82,2–85,0	1,14	1,11–1,17
>2 e ≤ 3 SM	87,7	85,3–89,8	1,26	1,21–1,31	88,2	86,2–90,0	1,2	1,17–1,24
> 3 SM	90,0	88,0–91,7	1,29	1,25–1,34	91,6	89,9–93,0	1,25	1,21–1,28
Região								
Norte	75,0^a^	72,3–77,6	1	–	79,0^a^	77,2–80,7	1	–
Nordeste	74,2^a^	72,5–75,9	0,99	0,95–1,03	76,4^a^	75,2–77,5	0,97	0,94–0,99
Sudeste	80,4^a^	78,7–82,0	1,07	1,03–1,12	84,1^a^	82,7–85,3	1,06	1,04–1,09
Sul	82,6	80,2–84,7	1,1	1,05–1,15	84,8	83,3–86,2	1,07	1,04–1,10
Centro-Oeste	80,7	78,8–82,4	1,07	1,03–1,12	78,8	76,6–80,9	1	0,96–1,03
Área geográfica								
Urbana	79,6^a^	78,5–80,6	1,09	1,05–1,13	82,2^a^	81,4–83,0	1,1	1,07–1,13
Rural	73,0	70,5–75,4	1	–	74,8	73,0–76,4	1	–
Localização								
Capital	83,1^a^	81,9–84,2	1,08	1,05–1,10	85,0^a^	84,0–86,0	1,06	1,04–1,08
Restante do estado	77,2^a^	76,0–78,4	1	–	80,1^a^	79,2–81,0	1	–
Plano de saúde								
Sim	89,6	88,1–90,9	1,2	1,18–1,23	91,2	90,1–92,2	1,18	1,16–1,20
Não	74,5^a^	73,3–75,7	1	–	77,5^a^	76,6–78,3	1	–
Relação sexual nos últimos 12 meses								
Sim	83,6^a^	82,6–84,6	1,35	1,27–1,43	86,0^a^	85,1–86,8	1,31	1,24–1,38
Não	62,1	58,3–65,7	1	–	65,9	62,5–69,1	1	–
Ficou grávida alguma vez na vida								
Sim	82,4	81,2–83,5	1,11	1,07–1,16	82,2	81,4–82,9	1,06	1,04–1,09
Não	73,9^a^	71,0–76,6	1	–	77,5^a^	75,7–79,1	1	–

IC95%: intervalo de confiança de 95%; SM: salário mínimo; RP: razão de prevalência estimada pela regressão de Poisson bivariada.

^a^ Valor de p < 0,05 do teste qui-quadrado de Pearson ajustado pela correção de Rao-Scott comparando as prevalências de 2013 e 2019.

Ainda com os resultados da Tabela 2, a comparação de prevalências de acordo com os fatores sociodemográficos e geográficos, entre 2013 e 2019, indica aumento na cobertura do exame de rastreamento em todas as regiões, com exceção da região Centro-Oeste e entre as moradoras da zona rural. Não há aumento nesse intervalo de tempo também para as que têm plano de saúde privado, para as que não tiveram relações sexuais nos últimos 12 meses e entre as que já engravidaram.

Em 2013, 40,9% das mulheres que realizaram o exame de Papanicolaou no SUS receberam o resultado em menos de um mês. Já, para 87,2% das que realizaram em serviços privados de saúde referiram ter recebido nesse prazo, sendo a diferença entre esses dois grupos estatisticamente significativa. A mesma situação se repetiu em 2019 (39,7% e 91,4%, respectivamente) (Tabela 3).

**Tabela 3:** Distribuição percentual (e respectivos intervalos de confiança de 95%) de mulheres de 25 a 64 anos que fizeram o exame preventivo de câncer de colo de útero nos últimos três anos anteriores à pesquisa por tempo de recebimentos do resultado do exame segundo realização do exame pelo SUS ou em serviços fora do SUS, Pesquisa Nacional de Saúde, 2013 e 2019.

Quanto tempo depois recebeu o resultado do exame	2013				2019			
	
	SUS^a^		Não SUS^a^		SUS^a^		Não SUS^a^	
			
	%	IC95%	%	IC95%	%	IC95%	%	IC95%
Menos de 1 mês depois	40,9	39–2–42,7	87,2	86,0–88,4	39,7	38,0–41,3	91,4	90,5–92,3
Entre 1 mês e menos de 3 meses depois	42,0	40,2–43,8	7,8	6,9–8,8	41,4	39,6–43,2	6,1	5,2–7,0
3 meses ou mais	8,7	7,6–10,0	2,3	1,8–3,0	9,8	9,0–10,8	0,4	0,2–0,7
Ainda não recebeu	6,3	5,5–7,3	2,1	1,6–2,7	6,4	5,7–7,1	1,5	1,2–1,8
Nunca recebeu	1,4	1,1–1,8	0,2	0,1–0,3	1,9	1,6–2,3	0,3	0,1–0,5
Não foi buscar	0,6	0,4–0,8	0,4	0,2–0,6	0,8	0,6–1,2	0,4	0,2–0,5

IC95%: intervalo de confiança de 95%.

^a^ Valor de p < 0,001 do teste qui-quadrado de Pearson ajustado pela correção de Rao-Scott comparando as prevalências SUS e não SUS em cada ano.

Apenas 1,4% das mulheres que realizaram o exame Papanicolaou no SUS em 2013 relataram nunca ter recebido o exame. No entanto, esse percentual foi ainda menor (0,2%) para aquelas que realizaram o exame na rede privada, novamente indicando diferença estatisticamente significativa entre SUS e não SUS (p < 0,001). Em 2019 essa diferença entre ter feito o exame e não ter recebido o resultado no SUS e fora do SUS se manteve (p < 0,001) (Tabela 3).

Os principais motivos apontados para a não realização do exame nas duas edições da PNS foram: “não achar necessário”, apontado por 45% das mulheres em ambas as pesquisas, e “nunca ter sido orientada a fazê-lo”, indicado por 20,6% das mulheres, em 2013, e 14,8%, em 2019. Ter vergonha ou nunca ter tido relações sexuais aparecem em seguida. Em 2013, ter dificuldade para marcar o exame aparece entre 3,8% das mulheres, mas em 2019 esse motivo não foi referido (Tabela 4).

**Tabela 4:** Distribuição percentual (e respectivos intervalos de confiança de 95%) do principal motivo para nunca ter feito o exame preventivo de câncer de colo de útero, Pesquisa Nacional de Saúde, 2013 e 2019.

Principal motivo para nunca ter feito o exame preventivo de câncer de colo de útero	2013		2019	
	
	%	IC95%	%	IC95%
Não acha necessário	45,5	41,8–49,2	45,1	41,6–48,6
Nunca foi orientada para fazer o exame	20,6	17,5–24,1	14,8	12,8–17,1
Tem vergonha	9,8	8,0–12,1	13,1	10,8–15,7
Nunca teve relações sexuais	7,2	5,5–9,3	8,8	6,7–11,4
Outro	6,7	4,7–9,5	2,7	1,8–4,0
Teve dificuldade para marcar a consulta	3,8	2,8–5,2	–	–
Não sabe quem procurar ou aonde ir	0,7	0,4–1,1	2	1,1–3,6
O tempo de espera no serviço de saúde é muito grande	1,6	1,1–2,5	2,7	2,0–3,6
O serviço de saúde era muito distante ou teve dificuldades de transporte	1,6	0,9–3,0	1,9	1,0–3,5
Tem dificuldades financeiras	1,2	0,8–2,0	2,1	1,4–3,2
Está marcado, mas ainda não realizou	0,7	0,4–1,2	1,4	0,9–2,1
O horário de funcionamento do serviço é incompatível com suas atividades de trabalho ou domésticas	0,4	0,2–0,8	2,7	1,8–3,9
Não conseguiu marcar consulta pelo plano de saúde	-	–	0,5	0,2–1,5
Fez cirurgia de retirada do útero/histerectomia	-	–	2,3	1,2–4,5

IC95%: intervalo de confiança de 95%.

Entre as mulheres pertencentes à população-alvo que referiram nunca ter feito o exame (9,7% em 2013 e 6,1% em 2019) a maior prevalência foi entre pardas e pretas, de menor escolaridade e renda, maior paridade, sem plano de saúde, residentes nas regiões Norte e Nordeste, fora da capital quando comparadas às que realizaram o exame nos últimos três anos (dados não apresentados).

## DISCUSSÃO

Com base nas informações autorreferidas nas duas edições da PNS, a cobertura do exame de rastreamento para câncer do colo do útero (Papanicolaou) no Brasil aumentou entre 2013 e 2019 (78,7% e 81,3%, respectivamente; p < 0,01) entre mulheres de 25 a 64 anos de idade. Embora as coberturas tenham sido mais altas entre as brancas em relação às pardas e pretas, entre residentes das regiões Sul e Sudeste, entre as residentes de capitais e zonas urbanas, os maiores aumentos ocorreram entre as mulheres de cor da pele preta, entre as com renda familiar até dois SM e entre as que não têm plano de saúde. Menores coberturas foram vistas entre as que não tiveram relações sexuais nos últimos 12 meses e entre as que nunca engravidaram. A proporção de mulheres que pagaram pelo exame foi maior em 2019, tanto entre as que referiram ter plano privado de saúde como entre as que não tinham.

Os resultados encontrados reforçam os achados de estudos nacionais e internacionais que mostraram associação entre raça, renda e escolaridade e a realização de exames de rastreamento. Na Bélgica e Suíça foi relatada a existência de associação de renda e nível de escolaridade à realização do exame preventivo, com diferenças entre as que nunca realizaram o exame e as que estavam em atraso. Este último foi associado a idades mais avançadas, enquanto nunca ter realizado o exame foi associado à nacionalidade estrangeira^23^. Nos Estados Unidos também se identificou associação da não realização do exame a idades mais jovens, não ser branca, ter menor renda e escolaridade^24^.

O SUS mantém-se como principal responsável pelo rastreamento no país. Chama atenção, contudo, que entre as mulheres que realizaram o exame houve aumento daquelas que pagaram, tanto entre as usuárias exclusivas do SUS como entre as que tinham plano privado de saúde. Esse achado, embora indique dificuldade no acesso ao exame no SUS ou no setor privado, pode refletir maior conscientização sobre a necessidade de realizá-lo. Por outro lado, o aumento da realização nos últimos três anos na faixa etária inferior a 25 anos reforça a necessidade de fortalecer as diretrizes clínicas que definem como população-alvo a faixa etária de 25 a 64 anos^4^.

Como o rastreamento é realizado no país de forma oportunística, ou seja, sem convocação ativa de mulheres na faixa etária-alvo, seria esperado que a busca por assistência pré-natal aumentaria a captação de mulheres para o exame de Papanicolaou, aproveitando-se o contato com o serviço de saúde. De fato, em 2013 e 2019, coberturas maiores foram observadas entre as mulheres que ficaram grávidas uma vez na vida, o que reforça que o momento do pré-natal aumenta a oportunidade de realização do exame. Entretanto, o modelo oportunístico não facilita a capitação de mulheres que nunca foram rastreadas, tampouco o seguimento daquelas com alterações no exame de rastreamento^25^.

Entre os principais motivos para a não realização do exame descritos na literatura destacam-se o desconhecimento sobre a importância do exame, medo, vergonha e dificuldades de acesso aos serviços de saúde^14^. Neste estudo, o principal motivo para não realizar o exame foi o não considerar necessário (45,1%), seguido pela falta de orientação para realizá-lo (14,8%), indicando a necessidade de investimentos na educação em saúde, principalmente entre as mulheres de menor renda e escolaridade, grupo no qual se encontra a maior proporção das que nunca realizaram o exame. Em um programa de rastreamento organizar estratégias de comunicação e convocação de mulheres na faixa etária-alvo são etapas indispensáveis e facilitam a adesão.

Para garantir o êxito de um programa de rastreamento, além da alta cobertura do exame, é necessário que as etapas subsequentes de investigação diagnóstica e tratamento estejam bem organizadas e disponíveis na rede de atenção à saúde. A demora no recebimento do resultado pode provocar o desinteresse e insatisfação da mulher, além de comprometer o seguimento adequado daquelas que tenham alterações a serem investigadas^25^. Além disso, a qualidade do exame de rastreamento é fundamental para identificar as lesões precursoras do câncer de colo do útero.

Um dos critérios de qualidade observados pelo Programa Nacional de Qualidade em Citopatologia é a liberação dos laudos pelo laboratório em até 30 dias após o recebimento do material^26^. Tanto em 2013 como em 2019, a prevalência de laudos do exame preventivo recebidos em até 30 dias foi cerca de duas vezes maior entre mulheres que realizaram o exame pelo plano de saúde se comparadas as que o fizeram na rede SUS. Tal constatação expõe dificuldades e desarticulação entre os serviços assistenciais do SUS, condições que levam à baixa resolutividade do programa^27^.

O modelo oportunístico e o seguimento inadequado impedem que se alcance um impacto equivalente ao de outros países da América Latina, como é o caso do Chile^13^. Em São Paulo, a implantação de um programa de rastreamento organizado em um município foi capaz de aumentar a cobertura e a identificação de casos de câncer em estágios iniciais^28^. Experiências internacionais confirmam esse achado, podendo-se citar a experiência da Eslovênia, que desde a década de 1960 realizava ações de rastreamento oportunístico do câncer do colo do útero, e com investimento na implantação de um programa nacional de rastreamento ao fim da década de 1990, observou-se redução de cerca de 40% na incidência de 2003 a 2009^29^.

A organização do rastreamento também implica na redução das desigualdades de acesso. Em estudo realizado em países da Europa a condição socioeconômica esteve associada à participação no rastreamento oportunístico, mas não foi observada em programas organizados^30^. Nos Estados Unidos, o programa de controle do câncer desenvolvido pelo Centro de Controle de Doenças utilizou como estratégia priorizar mulheres que nunca haviam feito o exame ou estavam em atraso. Os resultados iniciais do programa mostraram que a taxa de lesões precursoras e de câncer identificada nesse grupo foi quase duas vezes maior que entre as mulheres rastreadas frequentemente^31^.

Algumas limitações do estudo merecem ser destacadas. As informações utilizadas foram coletadas em entrevista com base em questionário estruturado em duas pesquisas transversais a partir de autorrelato dos entrevistados, o que pode ser passível de viés de memória. Essas pesquisas, no entanto, foram realizadas com rigor científico visando garantir a qualidade e confiabilidade da informação^32^. A generalização dos dados da PNS é considerada segura^33^ e tem contribuído para o planejamento e monitoramento de ações de saúde no país.

No Brasil, a implantação de um programa de rastreamento com convocação ativa da população-alvo e monitoramento das ações deve ser prioridade da política de controle do câncer. É fundamental incluir mulheres das parcelas mais vulnerabilizadas da população, pois são elas as de maior risco de morrer por uma doença que pode ser evitável.

Os resultados deste estudo mostram que as coberturas do rastreamento do câncer do colo do útero no Brasil, apesar de serem relativamente altas, ainda expõem desigualdade no acesso e, principalmente, no recebimento do resultado em função do nível socioeconômico, cor da pele e ter plano de saúde privado. Apesar da ampliação do acesso observada para esses grupos, vários obstáculos precisam ser superados para que o rastreamento possa ter o impacto esperado na morbidade e mortalidade pela doença.
